# Juglone and 1,4-Naphthoquinone—Promising Nematicides for Sustainable Control of the Root Knot Nematode *Meloidogyne luci*

**DOI:** 10.3389/fpls.2022.867803

**Published:** 2022-05-17

**Authors:** Carla Maleita, Ivânia Esteves, Mara E. M. Braga, Joana Figueiredo, Marisa C. Gaspar, Isabel Abrantes, Hermínio C. de Sousa

**Affiliations:** ^1^Department of Chemical Engineering, Chemical Process Engineering and Forest Products Research Centre, University of Coimbra, Coimbra, Portugal; ^2^Department of Life Sciences, Centre for Functional Ecology – Science for People and the Planet, University of Coimbra, Coimbra, Portugal

**Keywords:** bionematicides, hatching, mortality, penetration, plant-parasitic nematodes, reproduction

## Abstract

The scarce availability of efficient and eco-friendly nematicides to control root-knot nematodes (RKN), *Meloidogyne* spp., has encouraged research toward the development of bionematicides. Naphthoquinones, juglone (JUG) and 1,4-naphthoquinone (1,4-NTQ), are being explored as alternatives to synthetic nematicides to control RKN. This study expands the knowledge on the effects of these natural compounds toward *M. luci* life cycle (mortality, hatching, penetration, reproduction). *M. luci* second-stage juveniles (J2)/eggs were exposed to each compound (250, 150, 100, 50, and 20 ppm) to monitor nematode mortality and hatching during 72 h and 15 days, respectively. Tomato seedlings were then inoculated with 200 J2, which had been exposed to JUG/1,4-NTQ for 3 days. The number of nematodes inside the roots was determined at 3 days after inoculation, and the final population density was assessed at 45 days after inoculation. Moreover, the potential mode of action of JUG/1,4-NTQ was investigated for the first time on RKN, through the assessment of reactive oxygen species (ROS) generation, acetylcholinesterase (AChE) *in vitro* inhibitory activity and expression analysis of *ache* and glutathione-S-transferase (*gst*) genes. 1,4-NTQ was the most active compound, causing ≥50% J2 mortality at 250 ppm, within 24 h. At 20 and 50 ppm, hatching was reduced by ≈50% for both compounds. JUG showed a greater effect on *M. luci* penetration and reproduction, decreasing infection by ≈80% (50 ppm) on tomato plants. However, 1,4-NTQ-induced generation of ROS and nematode vacuolization was observed. Our study confirms that JUG/1,4-NTQ are promising nematicidal compounds, and new knowledge on their physiological impacts on *Meloidogyne* was provided to open new avenues for the development of innovative sustainable nematicides.

## Introduction

To date, with 105 valid species described, the genus *Meloidogyne* [root-knot nematodes (RKN)] are among the top 10 plant-parasitic nematodes (PPN) in plant pathology with major ecological and economic impact worldwide (Jones et al., 2013; Ghaderi and Karssen, 2020). RKN are able to parasitize almost any species of plants, and due to their wide distribution in most agricultural areas all over the world, it seems understandable to estimate higher economic losses due to *Meloidogyne* species. Despite the lack of actual and global data on their real economic impact, Hussey and Janssen (2002) reported that every year, RKN are responsible for 5% of the crop losses, but this value is probably underestimated because plant symptoms caused by RKN are non-specific and losses are often attributed to other causes.

*M. arenaria, M. hapla, M. incognita*, and *M. javanica* are regarded as the most common RKN species; however, other species with apparently restricted distribution are considered species of emerging importance. *M. luci* is included in the Alert List of Pests of the European and Mediterranean Plant Protection Organization since 2017, as it represents a threat to the production of several important crops in European countries, where this species has been detected (Greece, Italy, Portugal, Slovenia, Turkey), but also in other parts of the world (Brazil, Chile, Guatemala, and Iran) (EPPO, 2017, 2021). Although *M. luci* has already been detected associated with economically important crops, no data are available on its real impacts on the quality and quantity of crop production. However, host suitability studies carried out in pots have shown that significant economic losses can be predicted due to *M. luci* (Maleita et al., 2018, 2022; Aydinli et al., 2019; Sen and Aydinli, 2021). In Portugal, *M. luci* was found the parasitizing roots of potato (*Solanum tuberosum*), tomato (*S. lycopersicum*), the ornamental plant *Cordyline australis*, and the weed *Oxalis corniculata* (Maleita et al., 2018; Santos et al., 2019; Rusinque et al., 2021).

Many strategies have been applied in RKN management. For the last 50 years, control of RKN largely depend on the use of synthetic nematicides, readily available in the market, rapid-acting, and highly reliable (Zasada et al., 2010; Desaeger et al., 2017). Nevertheless, many nematicides are being banned from the market or their use restricted (Nyczepir and Thomas, 2009; Johnson et al., 2012; Ebone et al., 2019). Concerns about the impacts of chemical nematicides on human health and the environment led many regulatory agencies, such as European Food Safety Authority (EFSA) and US Environmental Protection Agency (EPA), to strengthen pesticide regulation (European Commission, 2009a,b; Kopits et al., 2014). Today, the need for searching environmental friendly nematicides, deriving from plant extracts, posing low risks to humans or animals, is widely consensual (Haydock et al., 2006; Chen et al., 2020).

Several plant secondary metabolites (alcohols, aldehydes, fatty acid derivatives, terpenoids, and phenolics) have been identified as nematicidal compounds and/or with effects on second-stage juveniles (J2) hatching, paralysis and nematode root attraction, penetration, and reproduction (Aoudia et al., 2012; Ntalli and Caboni, 2012; Caboni and Ntalli, 2014; Aissani et al., 2015; Lu et al., 2017; Sikder and Vestergård, 2020).

Naphthoquinones (NTQ) are naturally occurring compounds in several families of plants, such as Ancistrocladaceae, Avicenniaceae, Balsaminaceae, Bignoniaceae, Boraginaceae, Dioncophyllaceae, Droseraceae, Ebenaceae, Gentianaceae, Iridaceae, Juglandaceae, Plumbaginaceae, Scrophulariaceae, and Verbenaceae, algae, fungi, lichens, and animals, such as beetles and arachnids (Raspotnig et al., 2005; Pankewitz and Hilker, 2008; Babula et al., 2009). Among NTQ, juglone (5-hydroxynaphthalene-1,4-dione; JUG) and 1,4-naphthoquinone (naphthalene-1,4-dione; 1,4-NTQ) were shown to have nematicidal activity, among other properties (insecticide, herbicidal, anti-inflammatory, antibacterial, antifungal, anticancer, and cytotoxic properties) and thus are promising compounds to develop novel, natural, and effective nematicides, with lower environmental half-lives than traditional nematicides (Babula et al., 2009; Fischer et al., 2012; Esteves et al., 2017; Maleita et al., 2017; Wang et al., 2017; Cha et al., 2019; Laxmikant, 2019; Aminin and Polonik, 2020; Islam and Widhalm, 2020). However, knowledge on the effects of these compounds through RKN life cycle and their potential mode(s) of action are still unknown. A possible mode of action is the inhibition of the acetylcholinesterase (AChE) enzyme, which is responsible for the termination of impulse transmissions at cholinergic synapses within the nervous system by rapid hydrolysis of the neurotransmitter acetylcholine. When it is inhibited, there is an excessive accumulation of acetylcholine, leading to nematode hyperactivity, incoordination, and finally paralysis and death (Piotte et al., 1999). Considering this mechanism of action, AChE is the target of some pesticides, which inhibit the enzyme, such as carbamates and organophosphates (Al-Rehiayani, 2008). Therefore, assays should be conducted to analyze nematicidal effect of JUG and 1,4-NTQ and possible action on AChE enzyme. The *in vitro* inhibition potency of some widely used nematicides has been already evaluated to control PPN, such as *M. javanica, Heterodera avenae*, and *Tylenchulus semipenetrans*, pathogens that are damaging to a wide range of crops (Islam and Widhalm, 2020).

Another hypothesis is the generation of reactive oxygen species (ROS) as a consequence of the reaction of quinones with glutathione (GSH) (Inbaraj and Chignell, 2004). ROS are strong oxidizing agents that are associated with several toxic effects playing a significant role in cell death (Widhalm and Rhodes, 2016).

The objectives of the study were as follows: (1) to evaluate the effects of JUG and 1,4-NTQ on the *in vitro* mortality, hatching, penetration, and reproduction of *M. luci* on tomato plants and (2) to infer their mode of action through the assessment of ROS generation, AChE *in vitro* inhibitory activity and expression analysis of *ache* and glutathione-S-transferase (*gst*) genes.

## Materials and Methods

### General Experimental Procedures

Pure bioactive compounds, JUG and 1,4-NTQ (Sigma-Aldrich, purity >97 and ≥96.5% w/w, respectively), were solubilized in Tween® 80 (Sigma-Aldrich, suitable for cell culture) at 2,500 ppm aqueous solution to obtain final concentrations ranging from 20 to 2,000 ppm, depending on the bioassay. Each concentration of bioactive compounds was prepared individually. Water and Tween® 80 were used as the controls in mortality, hatching, penetration, and reproduction bioassays. All bioassays were repeated two times, except penetration and reproduction bioassays. Solutions were stirred for 3 days, at 37°C, at 100 rpm, away from light.

### Root-Knot Nematode Isolate

An isolate of *M. luci* obtained from potato roots in Sepins (Cantanhede), Coimbra, Portugal (Maleita et al., 2018) was maintained on tomato cv. Coração-de-Boi, in pots containing sterilized sandy loam soil and sand (1:1 v/v), at 25 ± 2°C in a growth chamber. The isolate identification was confirmed by esterase phenotype analysis (Maleita et al., 2018).

### Mortality Bioassay

*M. luci* egg masses were handpicked from infected roots and placed in a hatching chamber. Hatched J2 from the first 24 h were discarded and subsequent J2, from the second 24 h, were collected. From these, 20 nematodes were handpicked individually into excavated glass blocks containing 1 ml of each JUG/1,4-NTQ concentration (20, 50, 100, 150, and 250 ppm). Glass blocks were maintained in a moist chamber, in the dark, at 22°C. J2 mortality was monitored at 6, 12, 24, 48, and 72 h after exposure. At each observation time, nematodes not showing movements when touched with a bristle were transferred to water and considered dead if they still failed to react. Each treatment consisted of four replicates.

### Hatching Bioassay

*M. luci* egg masses were collected from infected tomato roots and eggs were extracted using 0.52% sodium hypochlorite (NaOCl; Acros Organics B.V.B.A.) solution (Hussey and Barker, 1973). A total of fifty eggs (≈100 μl) were transferred into a 10-μm sieve placed in a glass block, and 1.5 ml of each JUG/1,4-NTQ concentration (20, 50, 100, 150, and 250 ppm) was added. The concentration of JUG/1,4-NTQ 20 and 50 ppm solutions was confirmed by high-performance liquid chromatography (HPLC) (*data not shown*; Seabra et al., 2019). Nematode hatching was monitored daily for 3 days and afterwards at 2-day intervals for 15 days, and it was assumed that NTQ activity was preserved during the tested period. Each treatment consisted of four replicates.

### Penetration and Reproduction Bioassays

J2 were obtained as referred above for the mortality bioassay: hatched J2 from the second 24 h and thereafter were collected and stored at 4°C, until a maximum of 3 days. Before inoculation, *M. luci* J2 were exposed for 3 days to JUG/1,4-NTQ solutions of 20 and 50 ppm; water and 2,500 ppm Tween® 80 were included as controls. A number of forty-eight tomato cv. Coração-de-Boi plants (3 weeks old) were transferred to pots (50 cm^3^) containing autoclaved sandy loam soil, sand, and substrate (1:1:1, v/v), and each was inoculated with 200 *M. luci* J2 (initial nematode population density, Pi). Only the mobile J2 were considered to define the volume of nematode suspension to be inoculated. Then, 3 days after inoculation, four roots/treatment were removed, roots were washed, stained with fuchsin acid (Sigma-Aldrich, purity ≥70%, w/w), and the number of nematodes inside the roots was recorded. The remaining five tomato plants/treatment were transferred into new pots (150 cm^3^). At 45 days after inoculation, the plants were harvested, and the root systems were washed carefully. The number of galls and egg masses per plant was recorded and categorized using a 0–5 scale (0 = no galls, 1 = 1–2, 2 = 3–10, 3 = 11–30, 4 = 31–100, 5 ≥ 100 galls) (Taylor and Sasser, 1978). Eggs were extracted from each root system using a 1% NaOCl solution (Hussey and Barker, 1973), the final nematode population (Pf) was determined, and the reproduction factor (Rf = Pf/Pi) was calculated.

### Acetylcholinesterase Inhibitory Activity Assay

Hydrochloric acid (HCl, 37%, p.a.) was purchased from Carlo Erba. AChE (Type VI-S, 500 U/mg protein), 5,5-dithiobis[2-nitrobenzoic acid] (DTNB, ≥98%), acetylthiocholine iodide (AChI, ≥98%), ethanol (≥99.8%, p.a.), and tris(hydroxymethyl)aminomethane (Tris Buffer) were obtained from Sigma-Aldrich.

AChE inhibitory activity *in vitro* assay was performed according to the modified procedure described by Ellman et al. (1961), and as previously carried out by Gaspar et al. (2020a,b).

Briefly, DTNB (3 mM, 500 μl), AChI (15 mM, 100 μl), Tris–HCl buffer at pH 8 (50 mM, 275 μl), and the JUG/1,4-NTQ (100 μl) were added to a 1-ml cuvette. Compounds JUG/1,4-NTQ were solubilized in ethanol and Tris-HCl buffer (50:50, v/v) and homogenized. The concentrations were based on preliminary assays and were 100, 250, 500, 1,000, and 2,000 ppm for JUG and 200, 250, 500, 1,000, and 2,000 ppm for 1,4-NTQ. The enzyme AChE (0.28 U/ml, 25 μl) was then added to start the reaction, which was monitored for 5 min, at 25°C and 405 nm (UV–vis spectrophotometer, Jasco, Model V650) for the determination of the reaction rate. The AChE activity was calculated as a percentage of this velocity compared to the control (ethanol:Tris-HCl buffer, 50:50 v/v), and the inhibitory activity was calculated by subtraction. The blank was performed for each sample and concentration, and using all reagents, except the enzyme to reduce the possible NTQ interference in the measured absorbance. For each sample, the assay was conducted in triplicate, with five concentrations.

Additional studies with several concentrations of the surfactant Tween® 80 (100, 2,500, and 5,000 ppm) were also conducted to understand about its possible action on AChE activity, since it is used to prepare the JUG/1,4-NTQ solutions used in nematode bioassays.

### Reactive Oxygen Species Assay

Production of ROS was evaluated by microscopic observation. *M. luci* J2 were incubated at 22°C for 3 days to JUG/1,4-NTQ at 20, 50, 100, 150, and 250 ppm. Water and Tween® 80 were also included as controls. After exposure, nematodes were washed two times with sterilized water, pelleted (336 g for 2 min), and incubated for 40 min in 2′,7′-dichlorofluorescein diacetate 20 μM (Sigma-Aldrich) at 20°C. Then, the nematodes were washed two times with sterile water, transferred to a 24-well plate, and paralyzed by the addition of 10 mM sodium azide (Sigma-Aldrich) (Sun et al., 2017; Rangsinth et al., 2019). Nematodes were randomly photographed using a Zeiss Observer Z.1 inverted microscope (Carl Zeiss) equipped with an AxioCam HRm camera and Zen Blue 2012 software, using a N-Achroplan 5 × /0.15 or a LD Plan-Neofluar 40 × /0.6. All conditions within an experiment were processed simultaneously and imaging settings (exposure time) were conserved.

### Gene Expression Analysis

The expression of *ache* and *gst* genes encoding an enzyme responsible for the primary termination of cholinergic nerve impulse transmission and an enzyme related to protection against the plant defense, respectively, were evaluated.

Approximately 20,000 J2 were incubated in JUG/1,4-NTQ 20 ppm, at 25°C also in the dark. Water and Tween® 80 at 2,500 ppm were used as controls. After 24 and 72 h, J2 were concentrated by centrifugation for 2 min at 336 g, washed three times with sterilized water and two times with RNAse-free water, and stored at −80°C, until RNA extraction.

Total RNA were extracted from J2 of *M. luci* and isolated with TRIzol reagent (Invitrogen). Nematodes were homogenized in TRIzol reagent (Sigma-Aldrich) through six freeze-thawing cycles in TyssueLyser (liquid nitrogen; 37°C; and 30 s at 50 Hz, respectively). Afterward, the RNA was purified using the Direct-zol RNA kit (Zymo research), and any remaining DNA was digested using the TURBO DNA-free kit (Ambion). The concentration and purity of the RNA were determined in a Nanodrop 2000c spectrophotometer (ThermoFisher), and the samples were stored at −80°C.

The RNA was converted into cDNA by reverse transcription (RT) using the iScript™ Reverse Transcription Supermix Kit (Bio-Rad Laboratories) in a volume of 20 μl, according to the instructions, and the samples were stored at −20°C, until polymerase chain reaction (PCR) analysis. Primer sets used are described in Table 1. The GST reverse primer (GST_R) was designed after amplification, cloning, and sequencing of *gst M. luci* fragment with MIHA-GSTS-1f/r primers (Table 1) (Duarte, 2014). PCR were performed in a 25 μl volume containing 1× Taq reaction buffer, 1.5 mM MgCl_2_, 0.2 mM dNTPs, 0.4 μM each primer, 2.5 U of Taq DNA polymerase (Bioline), and 25 ng of nematode cDNA as a template. An internal control—β-actin—was included. The amplifications were carried out in an MJ Mini Thermal Cycler (Bio-Rad) using the following conditions: an initial denaturation at 94°C for 3 min; followed by 30 cycles of denaturation at 94°C for 30 s, annealing at 52 and 53°C for 30 s for *ache* and *gst*, respectively, extension at 72°C for 30 s, and a final extension for 10 min at 72°C. PCR were analyzed on a 1% agarose gel electrophoresis in 1× Tris-borate EDTA buffer stained with GreenSafe (NZYTech).

**Table 1:** Primer sets used in the PCRs.

**Enzyme name**	**Primer name**	**Primer sequence 5^**′**^ → 3^**′**^**	**References**
Acetylcholinesterase	AChE_F	AACCGCAATCCAGACAATTCTTAT	Cui et al., 2017
	AChE_R	TCTTCTTGGCCCAGTTCCTATTCG	
Glutathione S-transferase	GST_F	GAAAAATGGCCAGCCGAGAA	Duarte, 2014
	GST_R	GAAGGATTGCGCCGCTC	This study^a^
β-actin*^*b*^*	Actin_F	GATGGCTACAGCTGCTTCGT	Duarte, 2014
	Actin_R	GGACAGTGTTGGCGTAAAGG	

a *Designed after amplification, cloning, and sequencing of gst M. luci fragment with MIHA-GSTS-1f/r primers described in Duarte (2014).*

b *Positive control*.

### Statistical Data Analyses

Data on *M. luci* J2 mortality were converted to percentage cumulative mortality, corrected by Schneider Orelli's formula with reference to water, used as experimental control:


$$\begin{array}{ll} Cumulative\;mortality\\ = & \left(\frac{\%\;mortality\;in\;treatment\;\%\;mortality\;in\;control}{100\;\%\;mortality\;in\;control}\right)\times 100 \end{array}$$

The effects of JUG and 1,4-NTQ on mortality, hatching, penetration, and reproduction were compared in one-way analysis of variance (ANOVA) followed by *post-hoc* Fisher's least significant difference (LSD) statistical test. Data on J2 mortality, during exposure in JUG, and J2 penetration were transformed (logarithmic and square root, respectively) to fulfill the assumptions of ANOVA (normality and variance homogeneity). Even after transformation of data on effect of 1,4-NTQ on J2 mortality, the assumptions of ANOVA were not fulfilled. Nonetheless, data were compared in ANOVA followed by *post-hoc* Fisher's LSD statistical test. A parametric test seemed to be more robust than a non-parametric test. Statistical analyses of the data were performed using Statsoft Statistica, version 7 for Windows.

Data on J2 mortality (48- and 72-h observations) and hatching inhibition (15-day observation) were subjected to Probit analysis (Finney, 1971), using PriProbit 1.63 software, and the lethal concentrations causing 50% mortality and 50% hatching inhibition (LC_50_) calculated. The concentration of bioactive compound able to inhibit 50% of the AChE activity (IC_50_) was also computed.

## Results

### Mortality Bioassay

**Figure 1:**
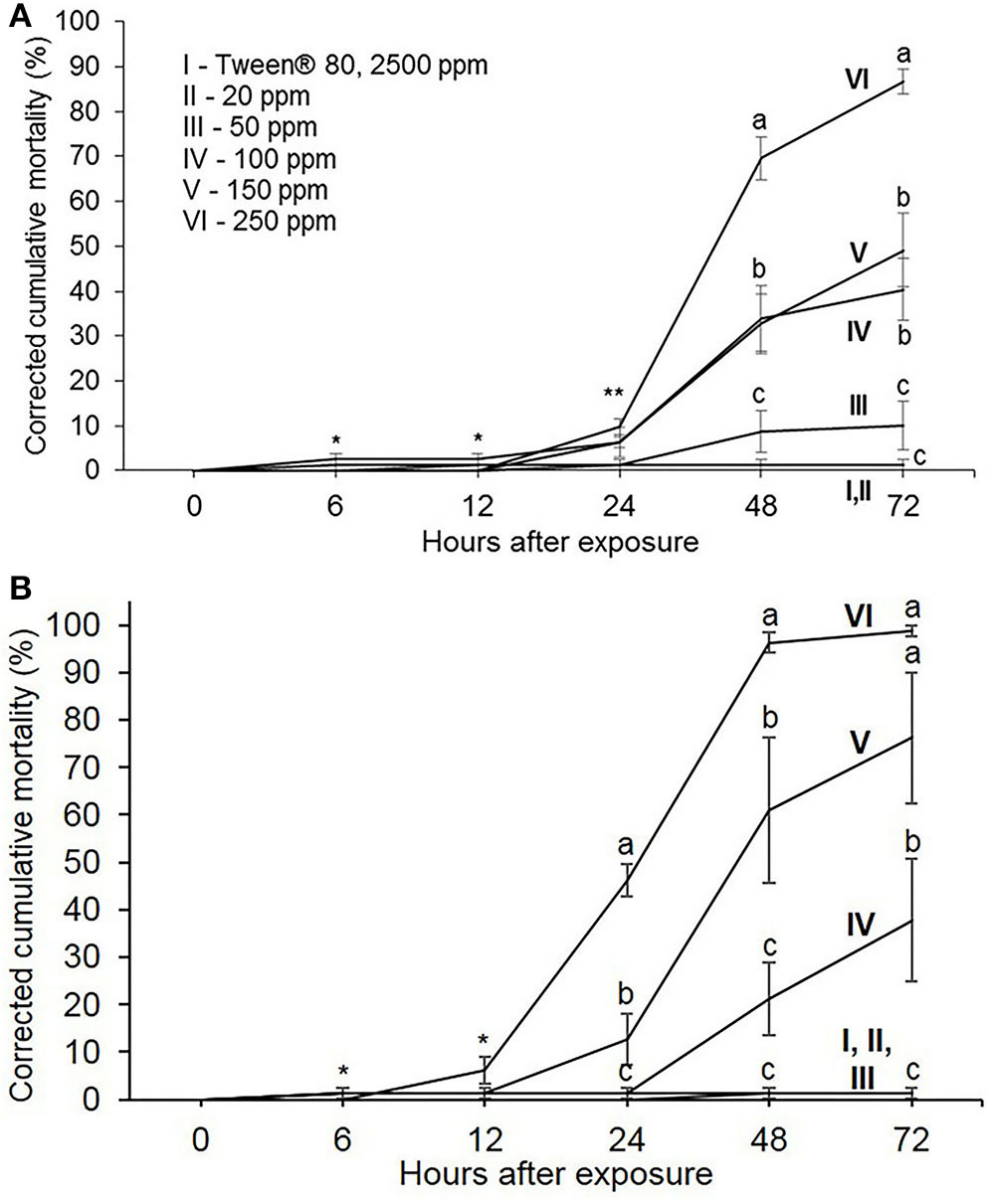
Corrected cumulative mortality (%) of *Meloidogyne luci* second-stage juveniles exposed to different concentrations of juglone (JUG, **A**) and 1,4-naphthoquinone (1,4-NTQ, **B**). Data are an average of four replicates, and bars represent standard errors. Average followed by the same lower case, at the same exposure time, do not differ significantly (*p* > 0.05) according to the Fisher's LSD test. ^*^At 6 and 12 h after exposure, no significant differences were found. ^**^IV and VI were significantly different from I, II, and III; V does not differ significantly from all treatments.

According to our experiments, *M. luci* mortality was significantly affected by the exposure to JUG and 1,4-NTQ (*p* > 0.05), for concentrations ≥100 ppm, and, in general, increases as compounds concentration increased. Nonetheless, compounds were not equally effective (Figure 1). 24 h after exposure to NTQ, JUG only induced 10% mortality, whereas 46% mortality was observed in 1,4-NTQ. JUG induced 32–70% mortality within 48 h, and 1,4-NTQ induced 61–96%, at 150 and 250 ppm, respectively (Figure 1). At 72 h after exposure, 1,4-NTQ induced 99% mortality and JUG 87%, at 250 ppm. A similar effect was detected for compounds at 100 ppm (38 and 40%, respectively) (Figure 1).

Estimated values of LC_50_, at 48 h after exposure, were 174.45 and 131.76 ppm to JUG and 1,4-NTQ, respectively, and 127.75 and 111.45 ppm at 72 h after exposure (Table 2). Therefore, 1,4-NTQ was more effective than JUG to induce *M. luci* mortality. At 24 h of exposure and for compound concentrations ≥100 ppm, most J2 remained alive but were immobile and only recovered the movement after being touched with a bristle. Dead nematodes showed, in general, a straight shape and vacuoles formation (**Figures 5, 6**).

**Table 2:** Estimated values of lethal concentration (ppm) necessary to result in 50% *Meloidogyne luci* second-stage juveniles' mortality and hatching inhibition (LC_50_), after exposure to juglone (JUG) and 1,4-naphthoquinone (1,4-NTQ).

**Bioassay**	**Time after exposure**	**LC**_**50**_ **(ppm)**	
		**JUG**	**1,4-NTQ**
Mortality	48 h	174.45	131.76
	72 h	127.75	111.45
Hatching inhibition	15 days	31.81	28.72

*M. luci* mortality in Tween® 80 (2,500 ppm), used for JUG and 1,4-NTQ solubilization, was not significantly different from that observed in water (control) and in the two NTQ, at 20 and 50 ppm, within 72 h after exposure (Figure 1).

### Hatching Bioassay

**Figure 2:**
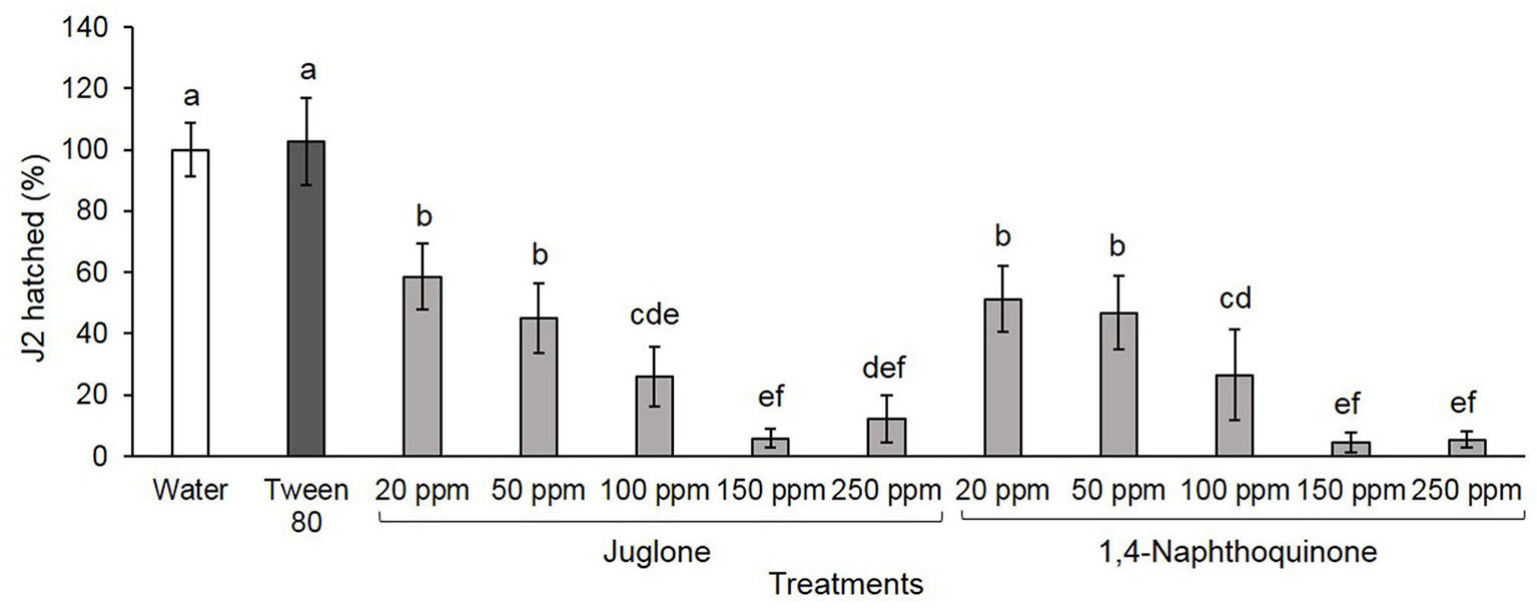
*Meloidogyne luci* second-stage juveniles (J2; %) hatched for 15 days. Eggs were soaked in water, 2,500 ppm Tween® 80 solution, and juglone and 1,4-naphthoquinone at different concentrations. Each bar represents the average ± standard deviation of four replicates and bars denoted by different letters differ significantly at *p* > 0.05, according to the Fisher's LSD test.

Both compounds have similar effects on hatching, inhibiting ≥49% J2 hatching within 15 days. At 20 and 50 ppm, J2 hatching was reduced by ~50% for both compounds reaching 96% at 250 ppm (Figure 2). In controls, water and Tween® 80 at 2,500 ppm (surfactant used to NTQ solubilization), 100% of *M. luci* J2, were hatched.

Estimated values of LC_50_, at 15 days after exposure, were 31.81 and 28.72 ppm to JUG and 1,4-NTQ (Table 2).

### Penetration and Reproduction Bioassays

**Figure 3:**
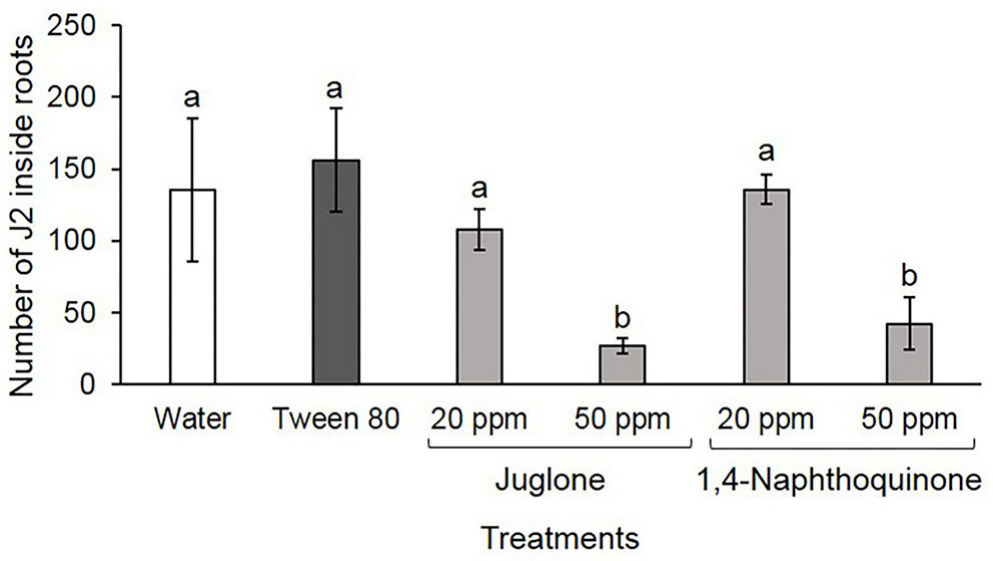
Number of *Meloidogyne luci* second-stage juveniles (J2) found inside tomato cv. Coração-de-Boi roots, 3 days after inoculation. Before inoculation, J2 were soaked for 3 days in water, 2,500 ppm Tween® 80 solution, and juglone and 1,4-naphthoquinone at 20 and 50 ppm. Each bar represents the average ± standard deviation of four replicates and bars denoted by different letters differ significantly at *p* > 0.05, according to the Fisher's LSD test.

Juglone arose as most efficient than 1,4-NTQ restricting significantly nematode root penetration and reproduction at 50 ppm. At this concentration, the number of J2 found inside the roots decreased ~69% for 1,4-NTQ and 80% for JUG when compared to water control. Penetration in water control was not significantly different from that observed in the Tween® 80 solvent solution and in both bioactive compounds at 20 ppm (Figure 3).

In terms of *M. luci* reproduction, 45 days after inoculation, in the water and Tween® 80 (2,500 ppm) controls, tomato cv. Coração-de-Boi root systems were highly infected with GI = 5 and Rf values of 268.5 and 344.8, respectively (Table 3). No significant differences were found between the controls. *M. luci* J2 developed and reproduced, after exposure to the bioactive compounds at 20 and 50 ppm, but the number of egg masses, Pf, and eggs/egg masses varied among treatments (Table 3). Although no significant effects were observed between the *M. luci* reproduction in water control and after exposure to 20 ppm of both compounds, the number of egg masses and Pf across treatments indicated significant differences between the bioactive compounds at 50 ppm and both controls (*p* > 0.05; Table 3). The number of eggs/egg masses after exposure to JUG 50 ppm decreased significantly, in ~32–33% when compared to water and Tween® 80 (2,500 ppm) controls (*p* > 0.05; Table 3).

**Table 3:** Number of galls (G) and egg masses (EM) and respective indices (GI, EMI), final population density (Pf), number of eggs/EM, and reproduction factor (Rf) of *Meloidogyne luci*, 45 days after inoculation^a^.

**Treatment**		**G**	**GI^**b**^**	**EM**	**EMI^**b**^**	**Pf**	**Eggs/EM**	**Rf^**c**^**
Controls	Water	>100	5	76.8 ± 8.3b,c	4	53,707 ± 10,604a,b	694.3 ± 77.7a	268.5
	Tween® 80	>100	5	97.6 ± 15.1a	4	68,967 ± 13,794a	704.0 ± 71.5a	344.8
JUG	20 ppm	>100	5	63.8 ± 17.5c	4	39,850 ± 13,306b,c	620.9 ± 85.7a	199.3
	50 ppm	>100	5	22.8 ± 7.5e	3	10,893 ± 4,166d	470.8 ± 87.5b	54.5
1,4-NTQ	20 ppm	>100	5	93.0 ± 18.3a,b	4	66,467 ± 16,677a	710.5 ± 81.1a	332.3
	50 ppm	>100	5	41.4 ± 5.4d	4	26,825 ± 8,509c	635.6 ± 151.6a	134.1

a *Data are means of five replicates ± standard deviation. Means in each column followed by the same combination of letters do not differ significantly at p > 0.05, according to the Fisher's LSD test.*

b *GI and EMI (0–5): 0 = no galls/egg masses, 1 = 1–2, 2 = 3–10, 3 = 11–30, 4 = 31–100, 5 ≥ 100 galls/egg masses per root system.*

c *Rf = Pf/initial population density (200 second-stage juveniles)*.

### Acetylcholinesterase Inhibitory Activity Assay

**Figure 4:**
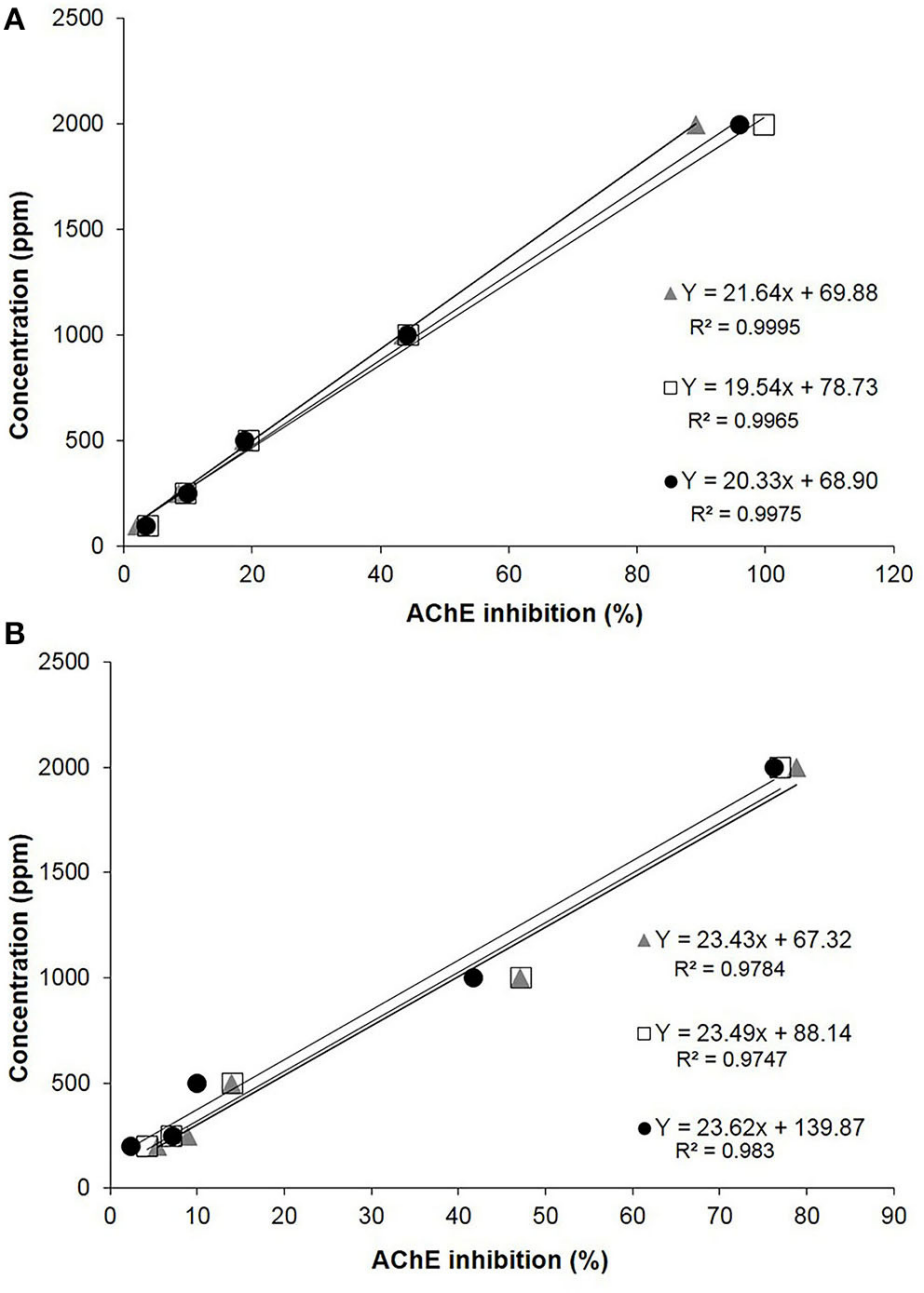
Acetylcholinesterase (AChE) inhibitory curves and respective *R*^2^ values obtained for juglone **(A)** and 1,4-naphthoquinone **(B)**.

Considering the effect of JUG and 1,4-NTQ on AChE activity, both compounds had similar inhibition of this enzyme activity, for concentrations over 200 ppm, and no effects are expected at 20 ppm. Results are expressed as IC_50_ values and AChE inhibitory curves are presented in Figure 4. The ethanol:Tris-HCl (50:50, v/v) buffer solution did not inhibit the AChE activity, and therefore, it was used for the solubilization of compounds.

For JUG, the IC_50_ was 1,097 ± 50 ppm, while for 1,4-NTQ, it was 1,274 ± 42 ppm. Considering the AChE inhibitory curves and the IC_50_ values, JUG was slightly more active than 1,4-NTQ, and both compounds showed inhibition of this enzyme activity, between 200 and 2,000 ppm, with values close to 100% inhibition for JUG at the highest concentration (Figure 4).

Additional experiments were conducted to analyze the possible inhibitory effect of Tween® 80 on AChE activity, and about 4 and 10% inhibition were observed at 2,500 and 5,000 ppm, respectively. This means that the Tween® 80 (2,500 ppm) has very low inhibitory activity on AChE, and it may be used in the assays, with caution and always performing the control (Supplementary Material).

### Reactive Oxygen Species Assay

**Figure 5:**
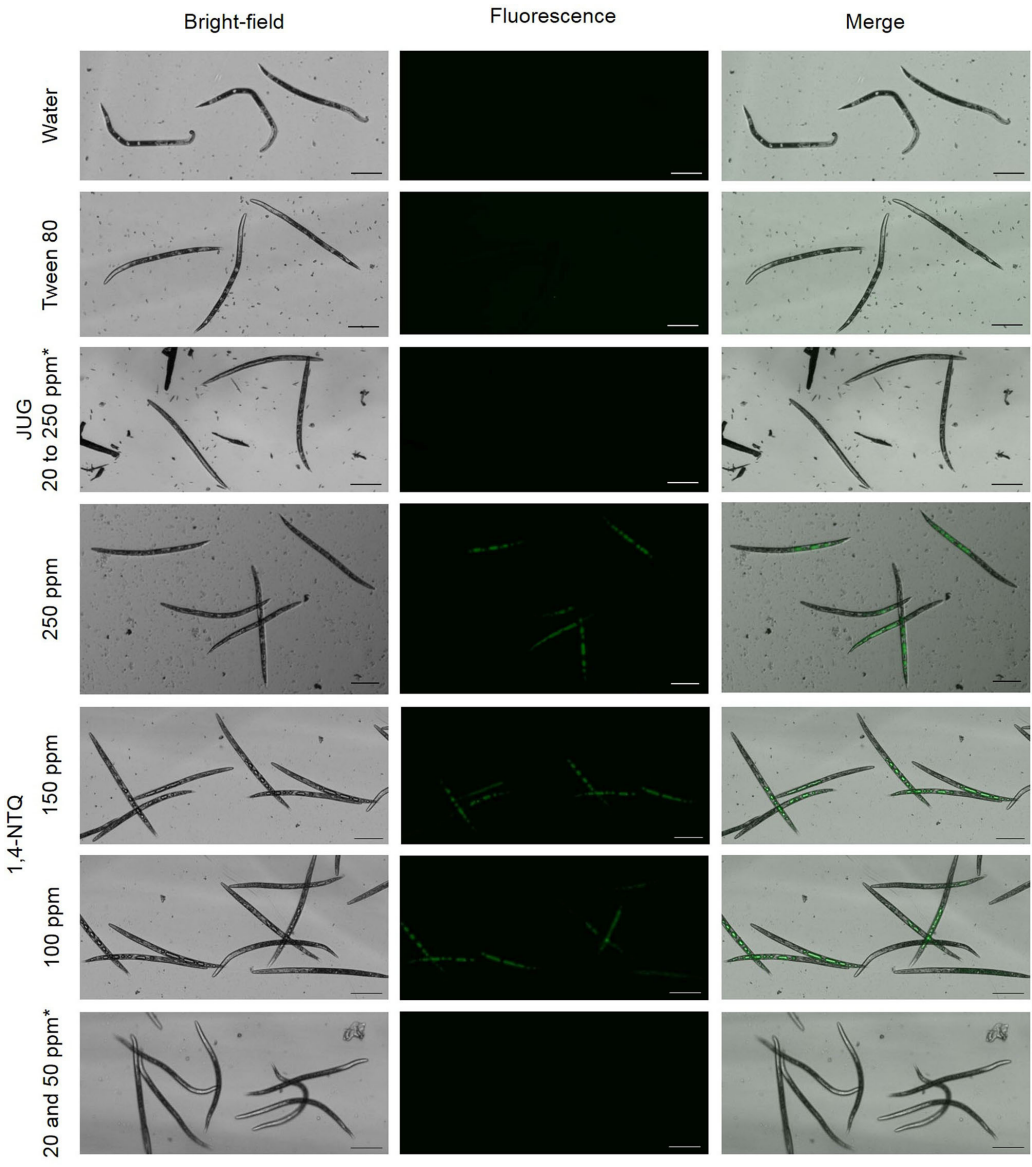
*In vitro* production of reactive oxygen species in *Meloidogyne luci* second-stage juveniles, after exposure for 3 days to juglone (JUG) and 1,4-naphthoquinone (1,4-NTQ) at different concentrations. *Similar results were obtained for referred concentrations; images presented correspond to the higher concentration. Scale bars = 100 μm.

**Figure 6:**
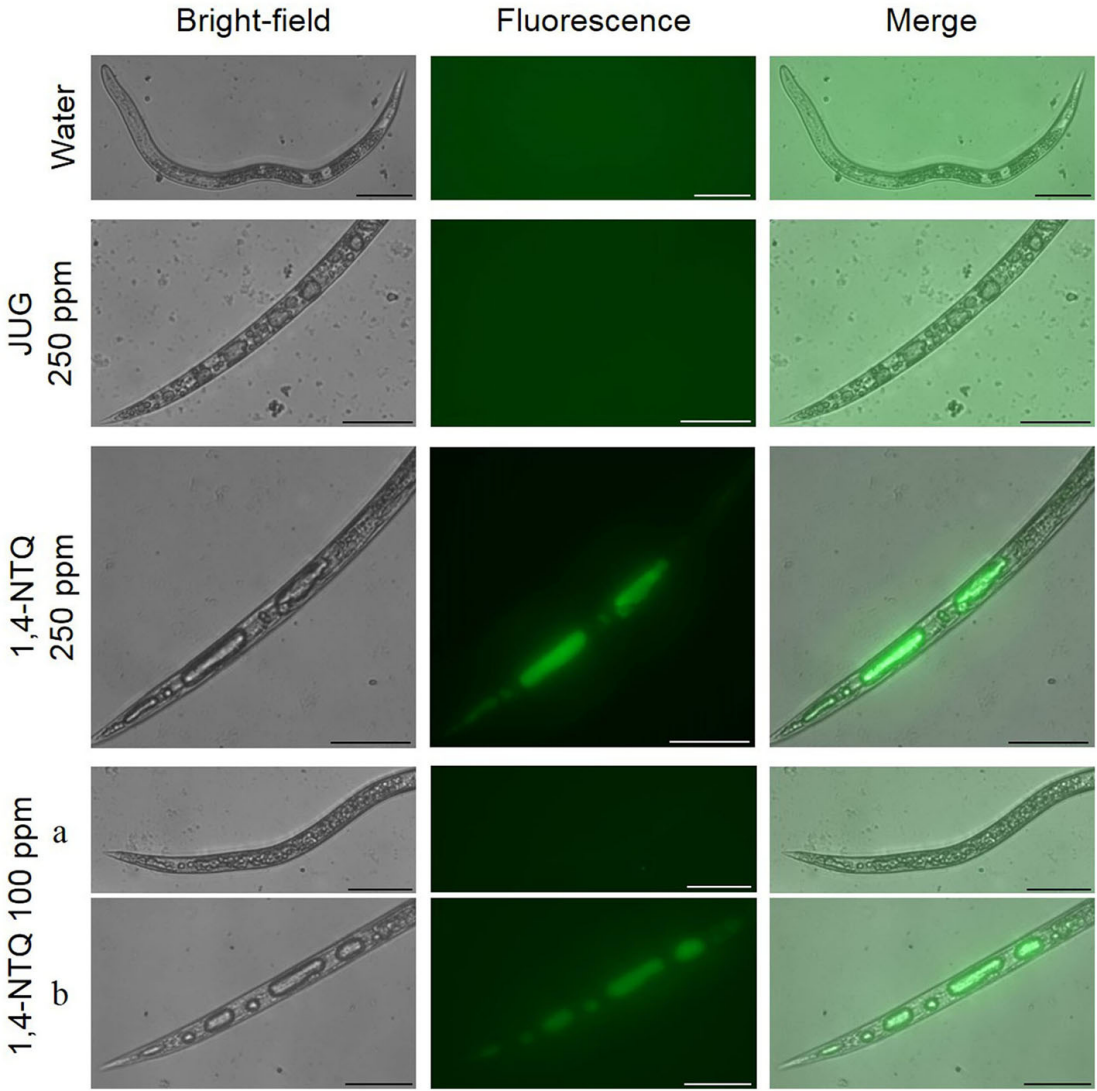
Effects of juglone (JUG) and 1,4-naphthoquinone (1,4-NTQ) on reactive oxygen species production in *Meloidogyne luci* second-stage juveniles' mortality. Nematodes were exposed to JUG and 1,4-NTQ at 100 and 250 ppm for 3 days. At 1,4-NTQ 100 ppm, some nematodes remained mobile **(a)** and others were dead **(b)**. Scale bars = 50 μm.

After nematode staining with 2′,7′-dichlorofluorescein diacetate (ROS indicator) and fluorescence monitoring, no J2 treated with water or Tween® 80 at 2,500 ppm (controls) generated ROS fluorescence or vacuoles development (Figures 5, 6). *M. luci* J2 treated with JUG did not exhibit ROS fluorescence but the formation of small and multiple vacuoles associated with mortality was observed whereas J2 treated with 1,4-NTQ exhibited ROS fluorescence for concentrations ≥100 ppm, and multiple giant vacuoles in the central and tail region were detected associated with nematode death. Several J2 exposed to 100 ppm 1,4-NTQ remained mobile and did not show vacuoles or ROS production (Figure 6,a).

### Gene Expression Analysis

**Figure 7:**
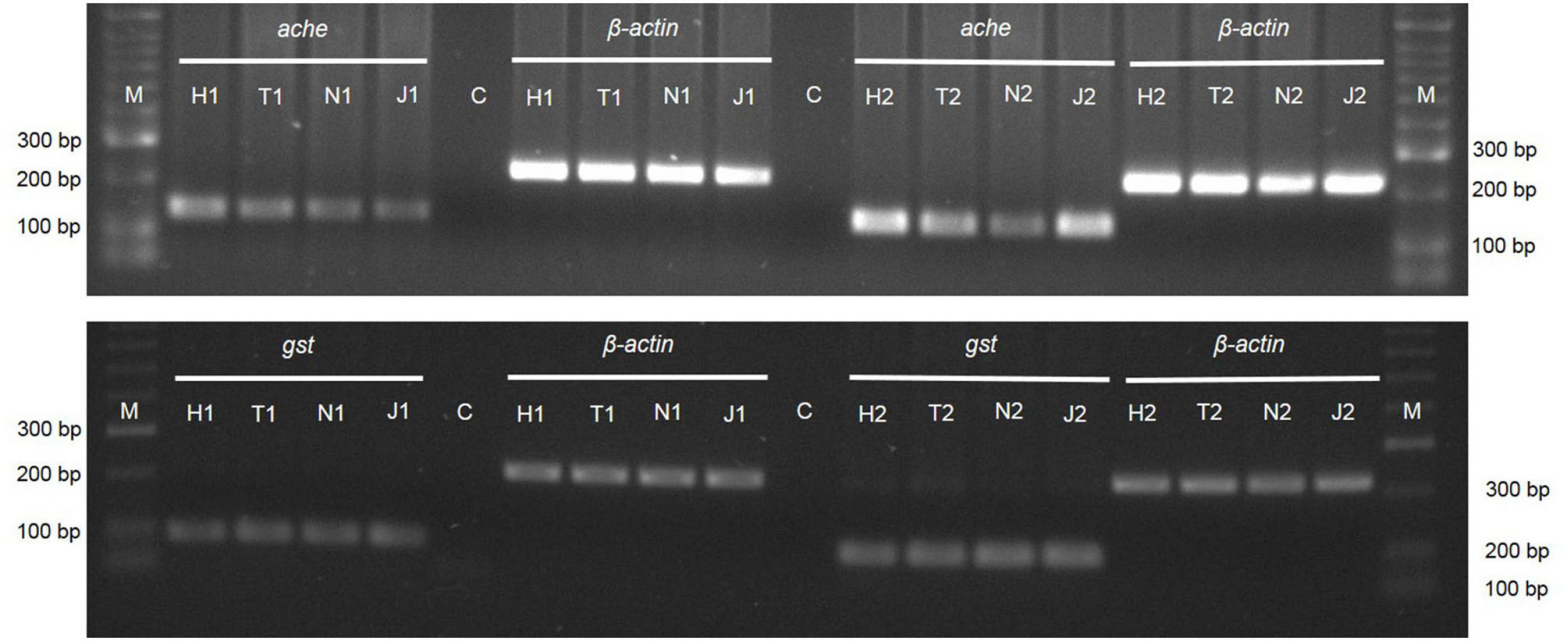
Expression of the genes acetylcholinesterase (*ache*), glutathione-S-transferase (*gst*) and β*-actin* by PCR amplification of cDNA from *Meloidogyne luci* second-stage juveniles after exposure for 24 h (1) and 72 h (2) to 1,4-naphthoquinone (N) and juglone (J) 20 ppm. Water (H) and Tween® 80 at 2,500 ppm (T) were used as controls. M - DNA Marker (HyperLadder II; Bioline).

Reverse transcription polymerase chain reaction, using the primers for *ache* and *gst* genes, displayed the expression of these genes in *M. luci* J2. Amplification of the β*-actin* gene was used as an internal control (Figure 7). The specific band for the *ache* gene has a molecular weight of ~150 bp and for the *gst* gene of 100 bp (Figure 7). The cDNA fragments of *ache* have a clear difference in band intensity after 24 h of J2 exposure to JUG and 1,4-NTQ and after 72 h of exposure to 1,4-NTQ when compared to those obtained in water. Tween® 80 (2,500 ppm) itself seems to slightly affect the *ache* gene expression. For *gst* and β*-actin* genes, the cDNA fragments were equally amplified in all treatments (Figure 7).

## Discussion

JUG and 1,4-NTQ were shown to be active against *M. luci*, inducing J2 mortality and inhibited hatching, penetration, and reproduction on tomato. Previously, few studies have been conducted to evaluate the *in vitro* and semi-*in vivo* nematicidal activity of these compounds against PPN (Mahajan et al., 1992; Dama, 2002; Esteves et al., 2017; Maleita et al., 2017; Cha et al., 2019; Laxmikant, 2019). According to Mahajan et al. (1992), JUG revealed a high degree of nematicidal activity promoting 100% *M. incognita* mortality at 1,100 ppm, within 48 h. Later, it was demonstrated that a concentration of 20 ppm JUG was efficient against *M. javanica* causing 97.94% mortality within 24 h (Dama, 2002). More recently, the nematicidal activity of JUG and 1,4-NTQ against the RKN *M. hispanica*, the root lesion nematode *Pratylenchus thornei*, and the pinewood nematode *Bursaphelenchus xylophilus* was proven (Esteves et al., 2017; Maleita et al., 2017; Cha et al., 2019). JUG and 1,4-NTQ 250 ppm caused 100% mortality in *M. hispanica* J2, after 24 and 12 h of exposure, respectively. 1,4-NTQ was most effective than JUG at 50 ppm, causing 42% *M. hispanica* J2 mortality (Maleita et al., 2017). Both compounds also induced >90% *B. xylophilus* mortality at 250 ppm, within 6 h and 100% *P. thornei* mortality within 24 h at 500 ppm (Esteves et al., 2017; Cha et al., 2019). Only Maleita et al. (2017) and Cha et al. (2019) studied the effects of these NTQ through semi-*in vivo* assays demonstrating the potential applications of these compounds in agriculture and forest industry. Incubation of *M. hispanica* J2 in a *Juglans nigra* extract, enriched in JUG and 1,4-NTQ, affected significantly nematode root penetration, and a reduction on reproduction was perceived (Maleita et al., 2017). The population of *B. xylophilus* decreased significantly, in *Pinus thunbergii* blocks, after treatment with JUG (Cha et al., 2019).

According to our experiments, 1,4-NTQ was most active than JUG on *M. luci* J2 mortality, despite being less effective against *M. hispanica* in the previous experiments (Maleita et al., 2017). Differences in the results obtained may be due to the use of a different surfactant (Triton X-100 vs. Tween® 80) to solubilize the bioactive compounds (Maleita et al., 2017) or a different sensitivity of these RKN species to the compounds. *M. incognita* isolates were reported as more sensitive to fluensulfone than *M. javanica*, whereas *M. javanica* was more sensitive to fluopyram, with a significant difference in median lethal concentrations (Oka and Saroya, 2019).

Both compounds have similar effects on hatching, but JUG arose as most efficient than 1,4-NTQ restricting significantly nematode root penetration and reproduction at 50 ppm. The NTQ, JUG and 1,4-NTQ, can be obtained from different plant species, such as *Pterocarya fraxinifolia, Carya* spp., *Lomatia* spp., *Caesalpinia sappan, Eleutherine palmifolia*, among others, and are found at higher concentrations in *Juglans* sp. tissues, depending on processing measures and solvent used for extraction (Borazjani et al., 1985; Lee and Lee, 2006; Solar et al., 2006; Jakopic et al., 2007; Cosmulescu et al., 2010, 2011; Widhalm and Rhodes, 2016; Maleita et al., 2017; Deans et al., 2018; Mahdavi et al., 2019; Annisa et al., 2020; Medic et al., 2021a,b). For example, Maleita et al. (2017) reported concentrations of 49.4 and 36.8 mg/g of extract of JUG and 1,4-NTQ, respectively, from *in natura J. nigra* with effects on *M. hispanica* J2 mortality and penetration; and McKenry and Anwar (2003) stated a reduction of 75% on *M. incognita* population, compared to the phenamiphos, after treatment of planted grapevines with a *Juglans* spp. tea.

Nowadays, pesticide registration is a very complex and highly regulated process. The risk assessment of JUG and 1,4-NTQ on the soil environment, which is an important source of concern for soil-applied nematicides, has been assessed previously for 1,4-NTQ (Chelinho et al., 2017). Although a dose–response was observed in plants (*Zea mays* and *Brassica napus*), non-target nematodes, and other soil invertebrates (*Eisenia andrei, Folsomia candida, Enchytraeus crypticus*), results showed that a concentration of ≈20 mg/kg 1,4-NTQ is likely to be environmentally safe (Chelinho et al., 2017).

Several studies have also been developed and reported the effect of JUG on the growth and development of several economically important plants, including aubergine (*Solanum melongena*), cabbage (*Brassica oleracea*), corn (*Zea mays*), cucumber (*Cucumis sativus*), lettuce (*Lactuca sativa*), pepper (*Capsicum annuum*), and tomato, to concentrations between 10 μM and 1 mM (Kocaçaliskan et al., 2019; Islam and Widhalm, 2020). For tomato crop, with potential yield losses of 25–100% due to RKN (Seid et al., 2015), concentrations of 1 mM JUG (Kocaçaliskan and Terzi, 2001) and ≥192 mg/Kg of JUG/1,4-NTQ (Maleita et al., *unpublished results*) caused a decrease in seed germination and seedling growth. The available results lead to consider that the JUG and 1,4-NTQ concentration needed to reduce *M. luci* penetration and reproduction (50 mg/kg) may be lower than the concentrations that negatively impact tomato development, and no significant effects are expected on non-target plants and soil organisms, including non-target nematodes.

To further develop the potential use of JUG and 1,4-NTQ as natural product-based nematicides, it is essential to improve the knowledge about the bioactive compound mode(s) of action. According to Inbaraj and Chignell (2004), the cytotoxicity of quinones is due to a redox cycling and reaction with GSH, resulting in the generation of ROS and decreasing the GSH intracellular levels which leads to significant overexpression of *gst* gene, respectively (Sytykiewicz, 2011). Glutathione transferases are a large family of enzymes involved in detoxification metabolism limiting oxidative damage of cellular macromolecules. These enzymes, produced by several organisms, are secreted by RKN during parasitism, protecting the parasite against ROS, but transcripts of the gene are also found in RKN J2 (Dubreuil et al., 2007).

The 1,4-NTQ has a general biological tendency to accept electrons originating highly reactive and unstable species that can be then auto-(re)oxidized, by molecular oxygen or by other chemical species, leading to the formation of the original NTQ and of highly ROS (Widhalm and Rhodes, 2016). Nevertheless, JUG may have pro- or antioxidant characteristics depending on the concentration, and generation of ROS can be not reported at lower concentrations due to the antioxidant activities of the compound (Chobot and Hadacek, 2009; Jha et al., 2015; Ahmad and Suzuki, 2019).

The vacuolization of *M. luci* J2 after exposure to 1,4-NTQ and the lack of fluorescence of nematodes exposed to JUG observed in this study suggest that the mode of action of the two compounds is probably different at 20 ppm. The 1,4-NTQ induced the formation of ROS and the development of multiple fused giant vacuoles inside nematodes; however, the transcriptional activity of *gst* was similar to the control. Glutathione-S-transferases are a large family of enzymes; therefore, further gene expression studies on a broader range of *gst* are needed to clarify the role of these enzymes on the metabolization of reactive compounds. Treatment with JUG 20 ppm induced the formation of multiple and small vacuoles, but no ROS formation was detected. Results may be related to the pro-antioxidant activity of JUG, which impedes the generation of ROS at lower concentrations. Consecutively, JUG may not influence GSH in RKN, and thus not inducing an overexpression of the *gst* gene.

Considering the effect of JUG and 1,4-NTQ on AChE activity, both compounds had similar inhibition of this enzyme activity, for concentrations over 200 ppm, and no effects are expected at 20 ppm. At a molecular level, a decrease in intensity of *ache* cDNA fragments at 24 h was found in nematodes exposed to 20 ppm JUG and 1,4-NTQ, compared to respective water control, which may indicate that, although at low concentrations of these compounds it is not possible to observe biological changes, the expression of these nematode genes seems to be affected. AChE is responsible for nematode nerve impulse transmission, and it is the predominant target of chemical nematicides used in agriculture against PPN, reducing the parasitic ability of the nematodes and impairing the life cycle completion (Combes et al., 2001; Cui et al., 2017). The results reveal that the 1,4-NTQ and JUG compounds slightly affect direct or indirectly nematode nerve impulse transmission, which was verified by J2 immobility, reacting only after being touched with a bristle. Nevertheless, multiple molecular forms of AChE were found in *Meloidogyne* spp., which specific functions were not identified in mutation studies, suggesting an overlapping function of the different classes (Chang and Opperman, 1991).

Therefore, the mode of action of these compounds on *M. luci* should be further investigated. Future studies on the *M. luci* transcriptome analysis after exposure to these NTQ will help to better understand the mode of action of these compounds on RKN.

In conclusion, the bioactive compounds JUG and 1,4-NTQ are very promising and attractive alternatives to the use of synthetic nematicides to control PPN, such as the RKN *M. luci*. The negative impact observed on hatching, root penetration, and reproduction of *M. luci* after exposure to JUG/1,4-NTQ at 50 ppm suggests that these compounds may be helpful in reducing the *M. luci* population in soil, contributing to limit the crop damage caused by this nematode to a level economically acceptable. Although 1,4-NTQ was shown to be most active than JUG on *M. luci* J2 mortality, low concentrations of JUG were efficient in restricting significantly nematode root penetration and reproduction. Thus, research on the development of nematicide formulations containing mixtures of both NTQ should be considered in the future. The development of bionematicide product based on NTQ compounds also opens the opportunity for valorization of agro-industrial byproducts, through the extraction of these compounds from walnut husk residues.

## Data Availability Statement

The original contributions presented in the study are included in the article/Supplementary Material, further inquiries can be directed to the corresponding author.

## Author Contributions

CM, IE, MB, JF, and MG: methodology. CM and HS: funding acquisition. CM: writing—original draft. All authors: contributed to the conceptualization, analysis, investigation, reviewing, and editing and read and contributed to the article and approved the submitted version.

## Funding

This research was carried out at the R&D Units Chemical Process Engineering and Forest Products Research Centre (CIEPQPF) and Centre for Functional Ecology—Science for People and the Planet (CFE), with references UIDB/00102/2020, UIDP/00102/2020 (CIEPQPF), and UID/BIA/04004/2020 (CFE), respectively, financed by Fundação para Ciência e a Tecnologia (FCT)/MCTES through national funds (PIDDAC) and at the European Regional Development Fund (FEDER) through the Programa Operacional Factores de Competitividade 2020 (COMPETE 2020) and national funds through FCT and FCT/MEC (PIDDAC), under contracts POCI-01-0145-FEDER-029392 and Centro-01-0145-FEDER-000007, CEECIND/02082/2017 (IE), CEECIND/000527/2017 (MG), and by Instituto do Ambiente, Tecnologia e Vida.

## Conflict of Interest

The authors declare that the research was conducted in the absence of any commercial or financial relationships that could be construed as a potential conflict of interest.

## Publisher's Note

All claims expressed in this article are solely those of the authors and do not necessarily represent those of their affiliated organizations, or those of the publisher, the editors and the reviewers. Any product that may be evaluated in this article, or claim that may be made by its manufacturer, is not guaranteed or endorsed by the publisher.
