# Supplementary material for: Juglone and 1,4-Naphthoquinone—Promising Nematicides for Sustainable Control of the Root Knot Nematode Meloidogyne luci
Source: Front Plant Sci. 2022 May 17;13:867803. doi: 10.3389/fpls.2022.867803 (PMC9152545; doi:10.3389/fpls.2022.867803)
Supplement: Supplementary file 1 [file Table_1.DOCX]

Supplementary Material

Three concentrations of Tween® 80 were tested in AChE *in vitro* assays, based on the use of this surfactant in other experiments: 100, 2500, and 5000 ppm. The control was performed, using water. About 10% inhibition was achieved for the highest tested concentration (**Figure S1**). The estimated IC_50_ value was 23016 ± 2170 ppm, but higher concentrations would be needed to calculate the value in a more accurate way. Nevertheless, this compound showed to have some degree of inhibitory activity on the AChE enzyme, and this was considered, because it could be associated to some results from other assays.


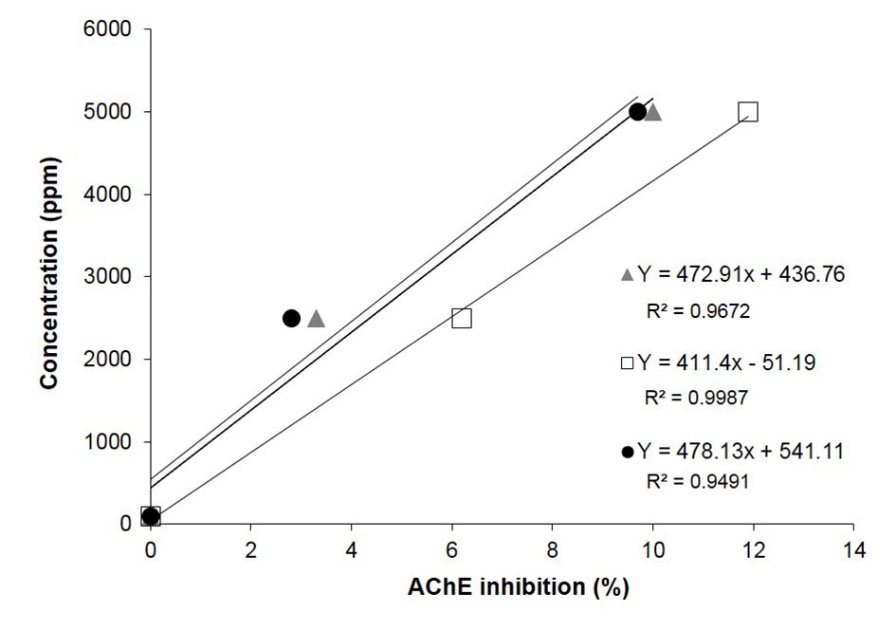


**Supplementary Figure 1.** Acetylcholinesterase (AChE) inhibitory curves and respective R^2^ values obtained for Tween® 80.
